# Supplementary material for: On the number of genealogical ancestors tracing to the source groups of an admixed population
Source: Genetics. 2023 Jul 6;224(3):iyad079. doi: 10.1093/genetics/iyad079 (PMC10324943; doi:10.1093/genetics/iyad079)
Supplement: iyad079_Supplementary_Data [file iyad079_supplementary_data.pdf]

Supplementary Information for "**On the number of genealogical ancestors tracing to the source groups of an admixed population**"

Epoch	Population	Minimum	Maximum
Epoch 1	European ($s_{2,1}$)	0	0.25
Epoch 2	African ($s_{1,7}$)	0	0.15
	African-American (h_7)	0.85	1.00
	European ($s_{2,7}$)	0	0.15
Epoch 3	African ($s_{1,10}$)	0	0.15
	African-American (h_{10})	0.85	1.00
	European ($s_{2,10}$)	0	0.15

Table S1. Ranges assumed for model parameters for a 3-epoch model of African-American demographic history. Note that in epoch 1, the African and African-American parameter values are generation-specific, set at $c_{g-1}(1 - s_{2,g-1})$ and $(1 - c_{g-1})(1 - s_{2,g-1})$, respectively, according to the values in Table 1. With an increment of 0.01 in the parameters, the total number of parameter sets considered is 480,896, the product of 26 choices for epoch 1 and $(16)(17)/2 = 136$ each for epochs 2 and 3. In epoch 2, 16 possible parameter sets are tested with $s_{1,7} = 0$, 15 with $s_{1,7} = 0.01$, 14 with $s_{1,7} = 0.02$, ..., 1 with $s_{1,7} = 0.15$; similarly, 16 sets are tested with $h_7 = 0.85$, 15 with $h_7 = 0.86$, 14 with $h_7 = 0.87$, ..., 1 with $h_7 = 1.00$, and 16 sets are tested with $s_{2,7} = 0$, 15 with $s_{2,7} = 0.01$, 14 with $s_{2,7} = 0.02$, ..., 1 with $s_{2,7} = 0.15$. Epoch 3 is comparable.

Generation g	Birth year	African ancestors		European ancestors	
		Mean	Standard deviation	Mean	Standard deviation
0	1610-1615	0.143	0.067	-	-
1	1635-1640	4.249	1.987	0.317	0.144
2	1660-1665	14.267	6.036	1.277	0.609
3	1685-1690	52.705	19.953	4.977	2.524
4	1710-1715	119.896	40.146	12.983	7.075
5	1735-1740	96.762	28.370	16.007	9.444
6	1760-1765	16.181	4.145	10.672	6.853
7	1785-1790	4.098	2.708	2.379	1.837
8	1810-1815	2.314	1.572	1.333	1.044
9	1835-1840	1.308	0.914	0.748	0.595
10	1860-1865	0.936	0.386	0.189	0.116
11	1885-1890	0.530	0.230	0.104	0.063
12	1910-1915	0.301	0.137	0.058	0.035
13	1935-1940	0.171	0.082	0.032	0.019
Total	-	313.859	102.769	51.076	18.736

Table S2. Generation-specific expectations of the numbers of African and European ancestors across accepted parameter sets. The table shows the values plotted in Figure 7.

		Probability of at least one ancestor	
Generation g	Birth year	African	European
0	1610-1615	0.0618	0.0000
1	1635-1640	0.8169	0.2970
2	1660-1665	0.9781	0.7230
3	1685-1690	0.9872	0.9661
4	1710-1715	0.9874	0.9857
5	1735-1740	0.9874	0.9865
6	1760-1765	0.9864	0.9844
7	1785-1790	0.9506	0.8795
8	1810-1815	0.8642	0.7251
9	1835-1840	0.7013	0.5280
10	1860-1865	0.6218	0.1694
11	1885-1890	0.4283	0.0986
12	1910-1915	0.2713	0.0563
13	1935-1940	0.1628	0.0317

Table S3. The probability of having at least one ancestor from a source population in a specified generation. The table shows the values plotted in Figure 8.

		Epoch 1	Epoch 2			Epoch 3		
Epoch	Parameter	$s_{2,1}$	$s_{1,7}$	h_7	$s_{2,7}$	$s_{1,10}$	h_{10}	$s_{2,10}$
3	$s_{2,10}$							
3	h_{10}							0.095
3	$s_{1,10}$						-0.972	-0.328
2	$s_{2,7}$					0.281	-0.217	-0.318
2	h_7				-0.361	-0.061	0.046	0.073
2	$s_{1,7}$			-0.708	-0.404	-0.153	0.118	0.169
1	$s_{2,1}$		0.336	-0.013	-0.427	0.430	-0.352	-0.402

Table S4. Pearson correlations of estimated parameters. The correlations are computed across all accepted parameter sets.

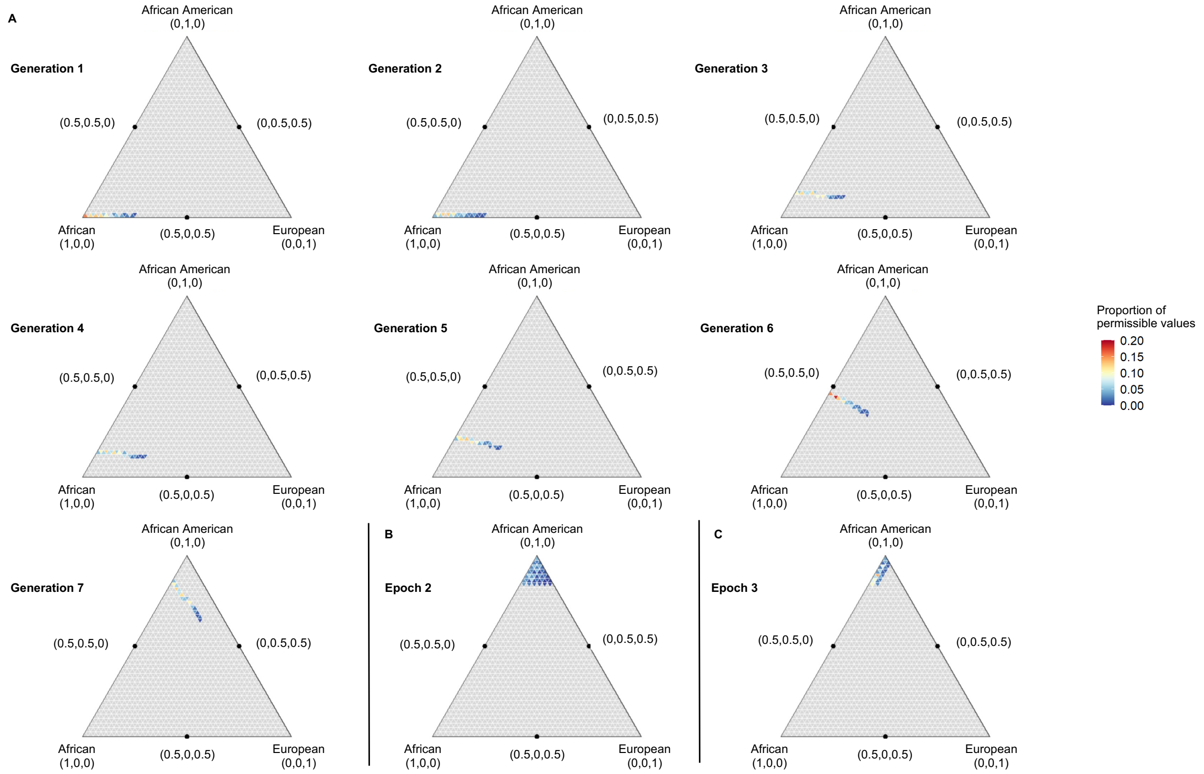

Figure S1. Distributions of generation-wise ancestry contributions estimated for African Americans, visualized in ternary plots. The figure shows a different visualization of accepted parameter sets plotted in Figure 4, illustrating the constraint that African, African-American, and European admixture contributions sum to 1. The permissible parameter sets follow Table S1. (A) Epoch 1. (B) Epoch 2. (C) Epoch 3.
